# Outcomes of the Integrated Scaling Approach: scaling an evidence-based employment intervention in public mental health systems

**DOI:** 10.3389/fpsyt.2026.1867975

**Published:** 2026-10-19

**Authors:** Marianne Farkas, Alexa Trolley-Hanson, Susan R. McGurk, Jane K. Burke-Miller, David Braverman, Chitra Khare, Kim T. Mueser

**Affiliations:** Center for Psychiatric Rehabilitation, Sargent College of Health and Rehabilitation Sciences, Boston University, Boston, MA, United States

**Keywords:** evidence-based practice, implementation science, mental health systems, scaling-up, supported employment

## Abstract

Evidence-based practices (EBPs) in supported employment (SE) improve vocational outcomes for individuals with serious mental health conditions, yet their implementation and sustainment at scale remain limited. *Thinking Skills for Work* (TSW), a cognitive remediation intervention, is an EBP that increases vocational outcomes for SE participants. This study evaluated the Integrated Scaling Approach (ISA), designed to increase adoption of EBPs, such as TSW, across service systems. A field trial of ISA was conducted across SE programs in a single U.S. state. ISA strategies addressed scale-up by building stakeholder engagement and staff-organizational-system capacity, across three phases (preparation, implementation, and sustainment). Using a concurrent mixed-methods design, quantitative data assessed TSW implementation, while qualitative interviews and focus groups examined facilitators and barriers to scaling and sustainment. Nine agencies, managing twelve SE sites, enrolled in the study. Eight completed all ISA phases. Nineteen staff achieved TSW certification, and sixteen completed a Train-the-Trainer program. Eighty-five clients received TSW during the trial. The state modified its evaluation process to monitor TSW implementation. Qualitative findings indicated that structured training, implementation team engagement, and intervention flexibility, facilitated adoption. Long term sustainment was not achieved due to workforce shortages, resource constraints, and the absence of sustained system-level support, exacerbated by the COVID-19 pandemic. The ISA promoted short-term adoption and workforce capacity for an evidence-based employment intervention across multiple organizations, but not long-term sustainment. These findings underscore the necessity of integrating long term system-level policies and funding mechanisms with implementation strategies to achieve sustainable scale-up of EBPs in mental health systems.

## 1 Introduction

Evidence-based practices (EBPs) in behavioral health are well-documented and available (1), yet despite decades of research, their uptake in mental health systems remains limited (2–4). Without deliberate efforts to scale new interventions, adoption often lags substantially behind the establishment of evidence, if it occurs at all (5–9). This implementation gap is particularly consequential in the domain of employment services for individuals with psychiatric disabilities.

Employment has long been recognized as a critical component of recovery for people with serious mental health conditions (10). A robust body of research links employment to improvements in overall well-being, self-esteem, and recovery (11–13), reduced psychiatric symptoms (12, 14), and decreased vulnerability to rehospitalization (15–17). Evidence-based supported employment (SE) models, particularly Individual Placement and Support (IPS), consistently outperform traditional vocational services in employment outcomes (18–20). Despite this, only a minority of SE programs implement EBPs, like IPS, limiting access to individuals who could benefit (4, 21). Specific processes that focus on scaling up EBPs, particularly those targeting employment, are needed to address this persistent service gap.

Across behavioral health, public health, and education systems, a common set of facilitators and barriers have been identified as influencing successful scale-up of EBPs (2, 22). These include (1): the readiness of interventions for implementation and scaling (2); the availability and adequacy of organizational or agency resources for supporting the EBP (3); workforce capacity; and (4) the presence of sociopolitical support for implementation and scaling. Intervention readiness includes manualization of EBPs, adaptation of materials and procedures to accommodate diverse contexts, and development of mechanisms to assess fidelity and outcomes at scale (23–25). Insufficient organizational resources, particularly financial and technical support, and inadequate workforce capacity are significant barriers to scaling employment-related EBPs in mental health settings (2, 26–28). Sociopolitical contexts also affect scale-up by shaping funding streams, regulatory requirements, and monitoring systems that facilitate or inhibit adoption and long-term sustainment of the EBP (29–31).

More recent research emphasizes that scaling up EBPs is a complex, dynamic process beyond addressing discrete implementation barriers (24). Large-scale implementation efforts often narrowly focus on service delivery capacity, with insufficient attention to generating demand for EBPs (32, 33). Successful scale-up requires generating demand for the EBP by promoting it and increasing awareness among providers, administrators, people with lived experience, and family members, making it a priority for investment and broader system uptake (2, 24, 33, 34).

Effective scale-up also requires a coordinated approach in which implementation strategies are deliberately selected and tailored to address barriers and leverage facilitators across the workforce, organizations, and systems of service delivery (35–37). While a range of discrete implementation strategies have been identified (e.g., workforce training, organizational capacity building) and several conceptual frameworks developed to guide implementation processes (e.g., Reach, Effectiveness, Adoption, Implementation, and Maintenance [RE-AIM (38);]; Consolidated Framework for Implementation Research [CFIR (39);]), few approaches integrate these into a comprehensive model for scaling EBPs across multiple organizations within a defined system or region.

These frameworks clarify that terms such as short-term adoption, implementation, and sustainment describe conceptually distinct outcomes rather than interchangeable indicators of program success. Proctor and colleagues’ taxonomy of implementation outcomes distinguish constructs such as adoption, or actions to try a new practice, from fidelity, penetration, and sustainability, which reflect practice maintenance over time (40). Such outcomes are not only conceptually, but empirically distinct, i.e., acceptability and adoption are typically achieved early in implementation, while sustainability is observed, if at all, much later (41). These temporal differences are consistent with phased implementation models such as the Exploration, Preparation, Implementation, and Sustainment (EPIS) framework, which similarly distinguish short-term implementation activity from the longer-term maintenance of a practice (42, 43).

### 1.1 The integrated scaling approach

The Integrated Scaling Approach (ISA) was developed to promote the uptake of EBPs across multiple organizations operating within a larger system (e.g., state). The ISA integrates four implementation domains individually shown to facilitate scale-up across agencies and service systems including: (1) enhancing demand for the EBP; (2) developing staff capacity to deliver the intervention; (3) increasing organizational (agency) capacity to support implementation; and (4) strengthening system-level capacity to sustain delivery over time (2, 22–24, 44, 45). Collectively, ISA strategies are intended to increase the prioritization, short-term adoption, implementation fidelity, and long-term sustainment through integration of EBPs within routine services across agencies or organizations.

The ISA requires attention to all four implementation domains across participating organizations, while allowing strategies within those domains to be utilized flexibly and tailored to the EBP and the organizational and system contexts in which it is implemented. ISA also integrates a suite of technologies including online learning platforms, project management and communication tools, fidelity tracking tools, and tools for sharing project information and resources. This structure enables coordinated support across multiple sites within a wide system or region, while providing tailored implementation strategies to meet unique organizational needs. The ISA implementation framework, domains, and example strategies are shown in **Figure 1**.

**Figure 1:**
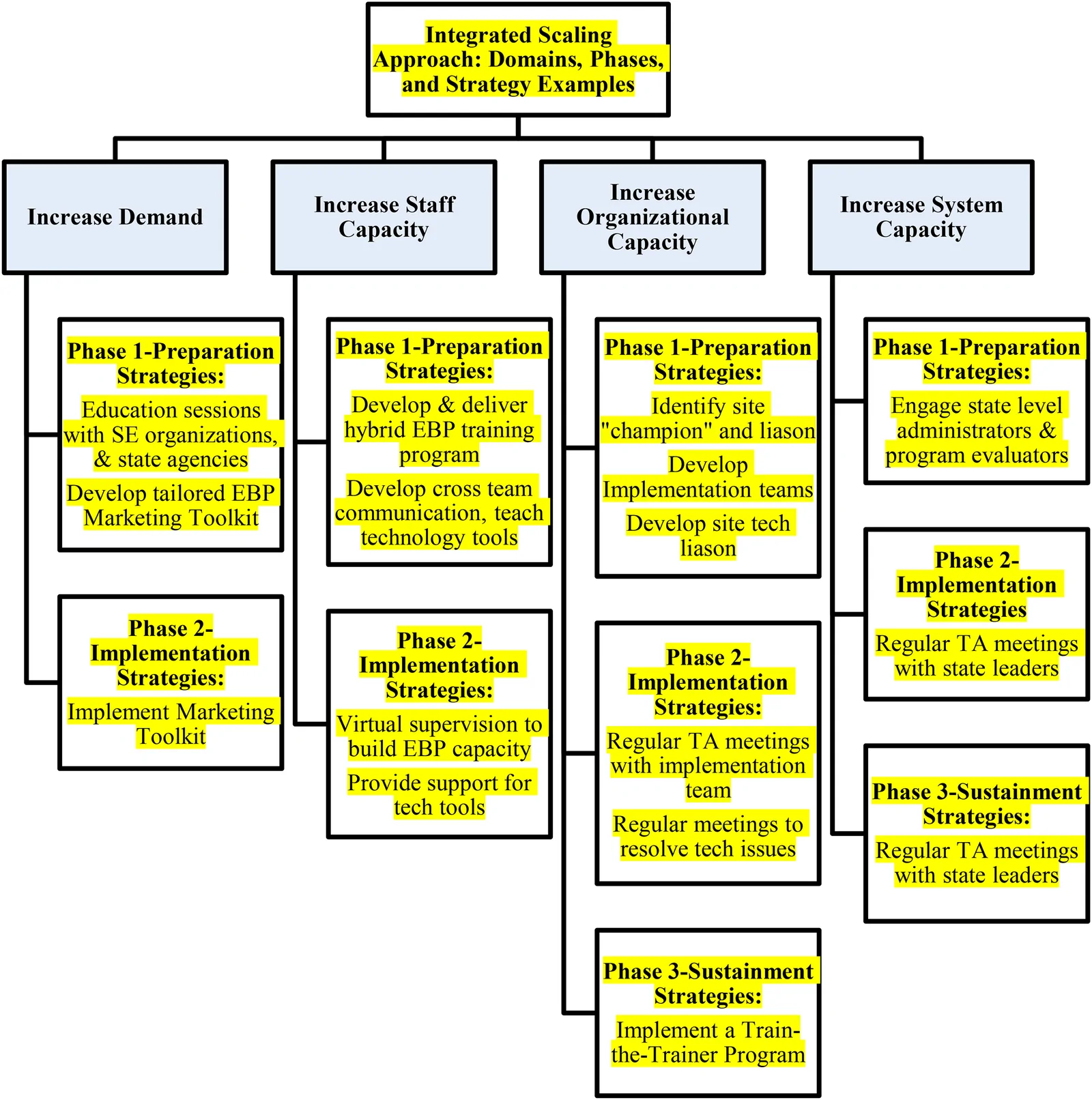
Integrated scaling approach. SE, Supported Employment; EBP, Evidence-Based Practice; TA, Technical Assistance.

The development of the ISA included an initial pilot project, in which individual ISA elements were evaluated in one SE agency in one state, followed by a secondary pilot that examined the integration of multiple elements across several SE agencies in another US state. The ISA was revised based on these pilot projects.

## 2 Methodology

### 2.1 The study

The purpose of the current study was to evaluate the finalized ISA through a 28-month field trial conducted across multiple SE agencies in a different U.S. state. The EBP selected for scale-up was Thinking Skills for Work (TSW) (**Figure 2**), a cognitive remediation intervention designed to improve employment outcomes among individuals with serious mental health conditions by enhancing cognitive functioning (19, 46, 47).

**Figure 2:**
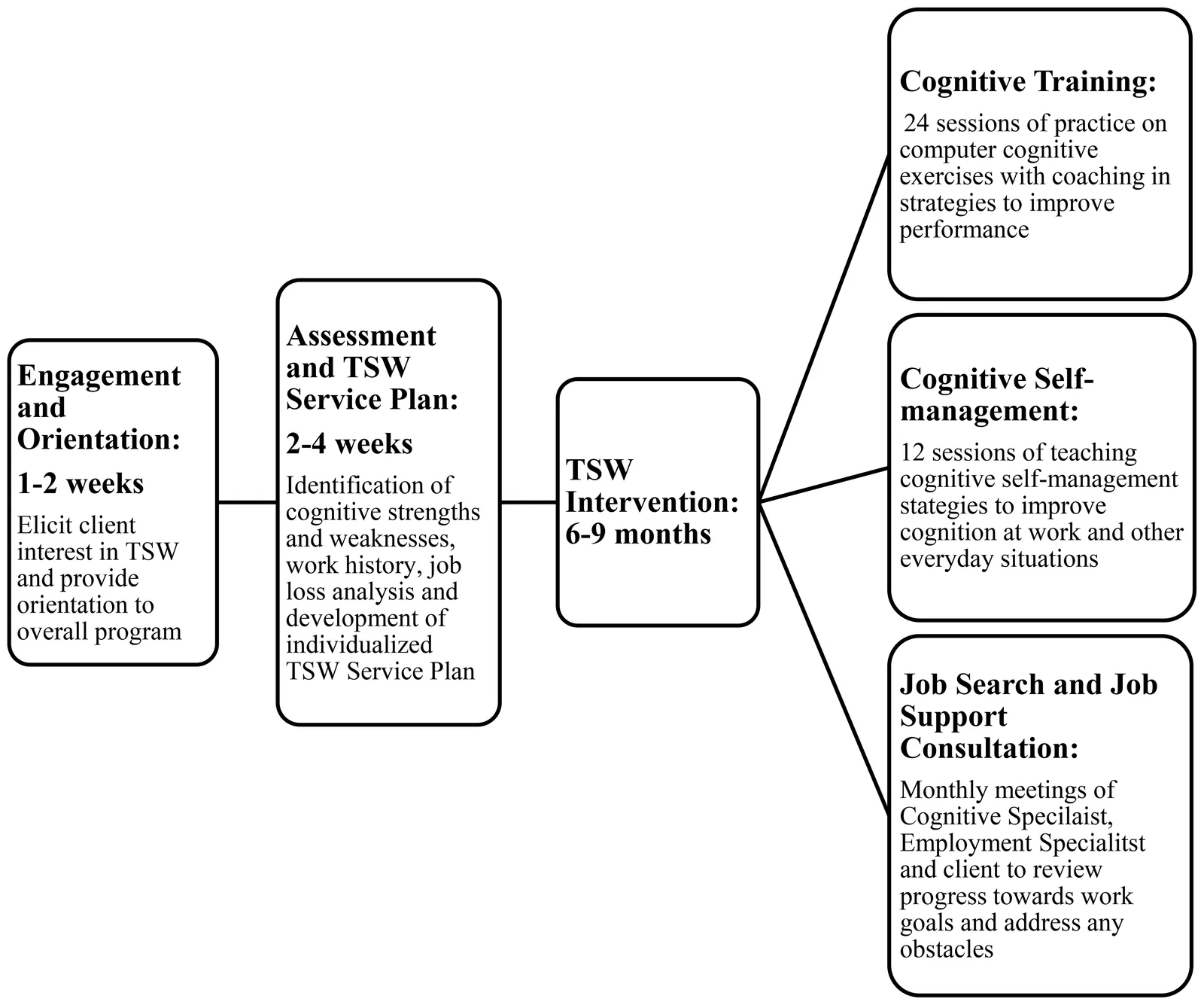
Thinking skills for work (TSW) program (52).

Research suggests that around 40-50% of participants with serious mental health conditions may not secure competitive work while enrolled in SE IPS programs (48). TSW was chosen as the EBP for the field trial as it addresses decreased cognitive functioning, a key barrier to vocational success in individuals with serious mental health conditions in SE programs (46, 49, 50). TSW also met ISA criteria for dissemination readiness, including a well-defined intervention manual and training guide (22).

#### 2.1.1 Study objectives

The objectives of this study were twofold: (1) to assess the effectiveness of ISA in facilitating the implementation of TSW across agency IPS programs within a state system; and (2) to identify facilitators and barriers to scaling TSW using the ISA framework.

### 2.2 Field trial: use of ISA & selected EBP

The field trial was conducted in (Redacted State, Georgia) which was selected based on the number of evidence-based SE programs and the size of the population served. At the time of recruitment, the state maintained 23 high fidelity IPS programs, collectively serving more than 4,000 individuals annually (51). Both the state Mental Health Authority (MHA) and the state Vocational Rehabilitation (VR) agency expressed interest in participating in the scaling initiative and engaging with the statewide SE network.

The ISA is organized into three phases of delivery: Preparation, Implementation, and Sustainment (see **Figure 1**). All three phases were applied in the Field Trial.

#### 2.2.1 Phase 1: preparation

Preparation phase activities focus on tailoring materials and technology to support implementation of the EBP, and building readiness across system, organizational, and stakeholder levels. In this study, these activities included engagement with state-level administrators to establish system-level support for the scaling initiative, and educational sessions with participating agencies to introduce TSW. Additionally, the field trial team adapted an online Marketing Toolkit to increase awareness of and demand for TSW among SE stakeholders.

Two implementation teams were created including (1) an agency level team composed of individuals with decision-making authority and frontline staff responsible for delivering TSW, and (2) a systems level team composed of state level stakeholders responsible for addressing the funding, regulation, and evaluation issues involved in scaling TSW.

Other ISA Preparation activities included the development and application of workforce training in the EBP. The field trial included the development and delivery of a 10-week, online, self-paced training program to establish service provider and supervisor knowledge of TSW. Finally, a suite of scaling-up technology tools was created to support those efforts by facilitating individual, group, and cross-group communication, project management, and delivery of education and training activities.

#### 2.2.2 Phase 2: implementation

The Implementation phase addressed TSW delivery and support. Technical assistance (TA) meetings were conducted regularly with agency implementation teams to support adapting their organizational infrastructure to incorporate TSW into daily practice (e.g., billing procedures, documentation practices, workflow integration). Concurrent TA meetings with the state level implementation team focused on establishing system-level support for TSW implementation, including mechanisms to monitor implementation and address coordination challenges.

SE providers participated in a TSW certification program which included five months of remote supervision that emphasized skill development, fidelity, and case-based problem-solving to ensure competency in the delivery of TSW.

#### 2.2.3 Phase 3: sustainment

Sustainment phase activities focused on supporting the long-term integration of TSW. While the ISA includes an array of potential sustainment strategies, the field trial focused on two strategies for the long-term integration of TSW, creating local training capacity and periodic TA. A 10-week Train-the-Trainer certification program enabled agencies to develop internal capacity to train new staff in TSW, mitigating staff turnover and reducing reliance on external training programs. Periodic TA meetings with agencies and state leaders provided opportunities to address organizational barriers and unanticipated challenges related to funding, leadership turnover, and policy alignment.

### 2.3 Data collection and measures

A concurrent mixed-methods design was employed to address the study aims. Quantitative data were used to assess the uptake of TSW across agencies (Aim 1), while qualitative data were collected to examine facilitators and barriers to scaling up (Aim 2).

#### 2.3.1 Quantitative measures

Quantitative data were collected by the research team, participating community agencies, and state partners throughout the project. Measures included: (1) the number of agencies participating in the ISA initiative; (2) the number of agencies completing all three ISA phases; (3) the number of staff completing TSW certification; (4) the number of clients receiving TSW services, as tracked by the state Mental Health Authority (MHA); and (5) the number of agencies and staff completing the train-the-trainer certification program.

#### 2.3.2 Qualitative measures

Qualitative data were collected through semi-structured focus groups and individual interviews with agency staff, supervisors, program managers, and representatives from the state MHA. Purposeful sampling was used to ensure that data were collected from all stakeholder groups and agencies. Interviews and focus groups were conducted remotely via Zoom by trained research staff using semi-structured interview guides. Sessions were video-recorded and transcribed for analysis.

### 2.4 Data analysis

Quantitative data were analyzed using descriptive statistics to summarize participation, training completion, and service reach. Qualitative data were analyzed using a directed content analysis approach (52, 53). A codebook, developed from the four ISA domains (intervention readiness, staff capacity, organizational capacity, and system capacity), was used to deductively code the transcripts. Two trained research assistants (A.T. & D.B.) read the transcripts for familiarity, then independently coded them using the established codebook while identifying new codes for emergent ideas. They met to compare codes, resolve discrepancies, and reach consensus. When conflicts arose, senior researchers (M.F. & K.M.) were consulted. Final codes were organized into themes of facilitators and barriers within each ISA domain and reviewed by the senior researchers (50).

## 3 Results

### 3.1 Aim 1: uptake of TSW use following ISA

Indicators of TSW use are summarized in **Table 1**. At the time of recruitment, all 23 SE agencies with IPS programs in the state were invited to participate in the field trial during the Preparation Phase. Of these, 12 agencies went on to submit applications, reviewed in terms of their baseline motivation to participate, (e.g. support for staff involvement in training; leadership willingness to participate in regular TA meetings, etc.). Nine agencies enrolled. One agency withdrew from the field trial during Implementation and stopped offering TSW to clients, however, they did have staff complete the train-the-trainer certification process. Eight agencies completed all three phases of the ISA (89%, 8/9).

**Table 1:** Integrated scaling approach (ISA) study aim 1: measures and results.

Study Aim 1: Assess the expanded use of TSW by SE teams following ISA	
Outcome measure:	Results:
Number of agencies within the state who participated in ISA	9 agencies with 12 sites (9/23 = 39% of active SE programs across the State).
Number of agencies who completed all ISA phases	8/9 agencies (89%) with 18 program managers participating in monthly TA sessions.
Number of staff who were certified in TSW	19 staff completed hybrid training and were certified.
Number of clients served as tracked by the state MHA	85 SE clients served (57 in phase 2 + 28 in phase 3).
Number of agencies/staff who completed the train-the-trainer certification process	16 staff at 9 agencies.

ISA, Integrated Scaling Approach; TSW, Thinking Skills for Work; SE, Supported Employment; EBP, Evidence-Based Practice; TA, Technical Assistance; MHA, Mental Health Authority.

During Implementation, 18 of 20 program managers (90%) participated in monthly TA sessions focused on TSW integration and 19 of 34 staff members (56%) across all agencies completed the training program and achieved TSW certification. During Sustainment, 16 staff members across all nine agencies completed the train-the-trainer certification program.

Also, during Implementation, agency providers identified and approached 61 clients as potential TSW participants, of whom 57 (93%, 57/61) enrolled and received services. During the Sustainment phase, an additional 28 clients were enrolled during resulting in a total of 85 SE clients receiving TSW during the field trial.

### 3.2 Aim 2: facilitators and barriers to scaling TSW using ISA

Twelve qualitative focus groups and two individual interviews were conducted with 25 participants, including frontline staff, supervisors, program managers, and one representative from the state MHA. VR withdrew from active participation during Implementation due to significant staff resignations during the COVID-19 pandemic, which co-occurred with the field trial, thus didn’t participate in the qualitative interviews.

Analysis identified multiple facilitators and barriers to scaling TSW, which were organized into themes by ISA domains: (1) stakeholder demand; (2) staff capacity; (3) organizational capacity; and (4) system capacity. These are summarized in **Table 2**.

**Table 2:** Integrated scaling approach (ISA) study aim 2: facilitators and barriers.

Study Aim 2: Identify facilitators and barriers to scaling up the delivery of TSW using ISA	
ISA strategy:	Facilitators:
Enhanced interest & demand	Use of a range of technology. Flexibility of TSW to optimize agency fit. Adaptability of TSW to meet specific client needs.
Staff capacity to deliver EBP	Highly structured, hybrid training programs focused on didactic learning and extended practice-based supervision. Staff skill competence development, enhancement of self-efficacy. Staff commitment to TSW based on positive experiences with professional development and client outcomes.
Organizational capacity to deliver EBP	Implementation team meetings promoted cross-agency learning, accountability, and problem-solving. Regular technical assistance meetings keep TSW visible amid competing demands. Project aims aligned with agency mission and values.
System capacity to support and sustain EBP	State support motivated agencies to participate. State leadership signaled legitimacy and potential value of TSW.
ISA strategy:	Barriers:
Enhanced interest & demand	Lack of client motivation exacerbated by COVID-19 pandemic.
Staff capacity to deliver EBP.	State-level funding reductions and workforce disruptions during the pandemic resulted in significant staff shortages and lack of capacity.
Organizational capacity to deliver EBP.	Agencies did not market TSW due to COVID-19 staff shortages. Staff were not trained due to COVID-19 staff shortages.
System capacity to support and sustain EBP.	No on-going funding mechanism developed due to State leadership and funding changes. No agency funding incentives for TSW due to State leadership change.

ISA, Integrated Scaling Approach; TSW, Thinking Skills for Work; EBP, Evidence-Based Practice.

### 3.3 Facilitators

#### 3.3.1 Stakeholder interest and demand for the EBP

Participants consistently described TSW as flexible and adaptable, facilitating its implementation across diverse agency contexts. Agencies reported being able to integrate TSW into existing SE workflows and tailor intervention components to meet individual client cognitive needs.

The use of technology including Zoom meetings, a dedicated TSW web portal, and project management software facilitated TSW training and monitoring facilitated the ISA knowledge dissemination strategies used in the delivery of TSW.

#### 3.3.2 Staff capacity to deliver the EBP

Interviewees emphasized how the structured hybrid training model, which combined didactic instruction with extended practice-based supervision, increased staff confidence and perceived competence in delivering TSW.

“I think we all thought that it was really easy to understand, easy to follow … It helped make sense of how it all fits together, and we felt much more confident when we were done.” (Participant 13).

Participants also highlighted perceived benefits to staff professional development and client outcomes, which reinforced their commitment to TSW implementation.

“After a while, I started seeing how much [TSW] was really assisting the individuals … it was equipping them in different areas.” (Participant 10).

The Train-the-Trainer program further strengthened staff capacity by preparing agencies to address workforce turnover and maintain internal expertise.

#### 3.3.3 Organizational capacity to deliver the EBP

Implementation team meetings were identified as a key facilitator of organizational readiness. These meetings promoted cross-agency learning, accountability, and problem-solving related to integrating TSW within SE services.

“It led to some really great discussions … about how [TSW] could be implemented in different programs.” (Participant 3).

Regular TA meetings helped prioritize TSW amid competing demands, reducing the likelihood that implementation efforts would lose momentum.

“If we didn’t have those regular meetings, it could have easily gotten lost.” (Participant 21).

#### 3.3.4 System capacity to support and sustain the EBP

State-level endorsement played an important role in facilitating agency participation. Participants reported that support from state leadership signaled the legitimacy and potential value of TSW, encouraging agencies to engage in the project.

“It was introduced to us in a state meeting … as another avenue for assisting the individuals that we serve.” (Participant 10).

### 3.4 Barriers

Participants identified several barriers that limited the implementation and sustainment of TSW, many of which were associated with the COVID-19 pandemic. Pandemic-related disruptions, such as significant reductions in staffing and funding, began between the Preparation and Implementation phases and affected nearly all aspects of the field trial.

#### 3.4.1 Stakeholder interest and demand for EBP

Participants described pandemic-related challenges to engaging clients in TSW, including clients prioritizing personal health over work, reluctance to engage in new interventions, and avoidance of in-person services.

#### 3.4.2 Staff capacity to deliver the EBP

Participants reported that state-level funding cuts and workforce disruptions during the pandemic resulted in significant staff shortages. Several agencies reported losing between one-third and one-half of their SE staff, limiting their ability to implement and sustain TSW.

“We’re in survival mode all the time … I don’t know that I gave TSW the attention it deserved.” (Participant 1).

#### 3.4.3 Organizational capacity to deliver the EBP

During the pandemic, agencies reported being unable to implement the Marketing Toolkit due to concerns that they would be unable to meet the demand for TSW services. Completion of TSW training was similarly affected, as staff were redeployed to cover other essential services (e.g., case management).

#### 3.4.4 System capacity to support and sustain the EBP

Participants identified the absence of a sustainable funding mechanism as a critical barrier to long-term sustainment of TSW. During the pandemic, the state’s VR system lost 75% of its staffing, as state funding was shifted to COVID relief, preventing them from providing vocational evaluations and participating in the field trial. Leadership and staffing changes at both MHA and VR disrupted plans to reimburse TSW as part of SE services, reducing the incentive for agencies to sustain TSW.

“Providers lost interest once they heard there was no additional funding … we can’t provide it for free.” (Participant 4).

These state-level changes halted proposed modifications to SE IPS fidelity measures that would have recognized TSW as a component of SE services, making it hard for some agencies to integrate TSW into the IPS model.

“The expectation is rapid placement … there’s no time to teach the individual.” (Participant 12).

## 4 Discussion

### 4.1 Summary of key findings

This study examined outcomes of using the Integrated Scaling Approach (ISA) to facilitate implementation and scaling of an evidence-based employment intervention, Thinking Skills for Work (TSW), within Supported Employment (SE) programs. The ISA was successful in increasing workforce competency, organizational readiness, and short-term adoption of TSW.

Eight of nine participating agencies completed all three phases of the approach, clients across all agencies received TSW, staff at all agencies achieved TSW certification, and internal training capacity was established through a Train-the-Trainer model. Nearly 40% (9/23) of the state’s SE programs implemented TSW, and 85 clients received the intervention during the field trial. Qualitative results suggest that a strong training program that included didactic training and technical assistance from EBP experts, created staff demand for the EBP and contributed to adoption, implementation, and interest in sustainment. Participants also stated how multi-level implementation teams, regular TA, and state regulatory involvement facilitated ISA outcomes. These outcomes demonstrate that a comprehensive, integrated scaling strategy incorporating supportive technology tools can successfully promote adoption and early implementation of an EBP across multiple organizations.

Despite high levels of engagement and initial implementation success, TSW was not sustained following the conclusion of the field trial. However, all nine participating agencies demonstrated the intent to offer TSW in the future by supporting staff to become in-house TSW trainers. This pattern is consistent with recent longitudinal evidence that early implementation outcomes do not reliably predict long-term sustainment, underscoring that these represent distinct outcome constructs rather than simply sequential stages of the same outcome (54).

Qualitative findings identified barriers including workforce turnover and limited organizational resources among the 8 remaining participating agencies. Most critically, the lack of stable sociopolitical support ultimately prevented agencies from being able to sustain TSW. Structural barriers such as changes in leadership buy-in and resources are well-established obstacles to successful implementation (2, 22–24, 44, 45), and were therefore specifically addressed in multilevel planning meetings with agencies and the State at the outset. The timing of the field trial, however, coincided with the onset of the COVID-19 pandemic, which exacerbated existing systemic structural weaknesses such as lack of access to technology, workforce shortages and fragile funding streams (55, 56). The pandemic affected ISA implementation at every level (client, provider, agency, system) and phase (Preparation, Implementation, Sustainment). As a result, the findings may be limited in their generalizability.

### 4.2 Impact of the COVID-19 pandemic

Pandemic-related funding shifts, staffing shortages, and service delivery disruptions within the state’s mental health and vocational rehabilitation systems led to shifts in priorities away from implementing new EBPs. Agencies reported greater client disengagement and loss of SE staff, while state systems prioritized crisis response and health-related services over workforce and employment initiatives (57).

These conditions limited agencies’ ability to integrate new practices into routine workflows and constrained their capacity to generate demand for TSW among clients and referral sources. At the system level, leadership turnover and staffing losses within both the Mental Health Authority (MHA) and Vocational Rehabilitation (VR) agencies halted planned efforts to establish reimbursement mechanisms and align SE fidelity expectations with TSW delivery. Without such policy and funding support, agencies lacked the incentives and resources necessary to sustain the intervention.

While many SE staff and administrators had not been familiar with the use of virtual technology in their day-to-day work, COVID-19 forced them to rapidly adopt them. The ISA demonstrated that with support, staff at every level were able to use a range of technologies to facilitate EBP implementation.

### 4.3 Study impact

Despite the unprecedented challenges posed by the pandemic, the ISA appeared to be a useful approach to scaling an EBP. The flexibility of the ISA allowed for a coordinated approach to the adaptation of the EBP to remote and hybrid training, staggered implementation timelines, and selective use of intervention components in response to workforce constraints. These features are consistent with emerging evidence that flexible, context-responsive strategies are critical to successful scale-up in complex service systems (24, 35–37).

The findings also highlight the limitations of scaling approaches that rely on organizational and workforce-level strategies in the absence of durable system-level support. Although the ISA addressed four core scaling domains (EBP demand, staff capacity, organizational capacity, and system capacity), system capacity proved to be the hardest to influence in this project. This underscores that sustained scale-up of EBPs in public systems depends on long-term policy, funding, and regulatory strategies that extend beyond individual agencies or implementation teams.

Given the demonstrated need for interventions such as TSW, the lack of its sustainment beyond initial successful implementation represents an opportunity truncated by the global COVID-19 crisis and the accompanying disruption to the socio-political context. Prior research suggests that approximately 40% of SE participants do not respond to standard services, often due to cognitive challenges (47, 49). In the study state, SE programs served more than 4,000 individuals annually, suggesting that up to 1,600 clients could potentially benefit from TSW. With sustained state level support (e.g. adequate funding, ongoing congruent policies and agency contracting), ISA-facilitated scale-up could substantially expand access to TSW.

### 4.4 Limitations

This study has two main limitations. First, the field trial was conducted in a single state, which may limit generalizability to other service systems with different funding structures or policy environments. Second, the extraordinary context of the COVID-19 pandemic complicates interpretation of both implementation and sustainment outcomes, as many identified barriers were intensified or directly caused by pandemic-related disruptions.

### 4.5 Future directions

Future research should examine the effectiveness of the ISA with different EBPs and in different systems under more stable system conditions. System level factors such as policies around reimbursement and regulation significantly affect the scaling-up and sustainment of EBPs. Future efforts should explore strategies for securing early and binding system-level commitments around such policies, to protect scaling initiatives from leadership turnover and shifting political priorities. Additionally, since marketing strategies had to be suspended due to the pandemic, future studies could explore the threshold point at which a lack of agency resources (like staffing shortages) negates the desirability of increasing the demand for EBPs.

## 5 Conclusion

The Integrated Scaling Approach offers a promising framework for promoting the adoption and implementation of EBPs across multiple organizations within public mental health systems. This study demonstrates that integrated strategies addressing workforce, organizational, and system-level factors can facilitate meaningful uptake, even during periods of significant disruption. However, the findings also reinforce a central finding in implementation research: without sustained sociopolitical and financial support for an EBP, even well-designed scaling efforts are unlikely to achieve long-term impact. Continued research and policy attention are needed to fully test the long-term effectiveness of the ISA in scaling up EBPs, like TSW, to meet the needs of more individuals with serious mental health conditions.

## Data Availability

The raw data supporting the conclusions of this article will be made available by the authors, without undue reservation.
